# Toward a Better Understanding of Species Interactions through Network Biology

**DOI:** 10.1128/mSystems.00114-19

**Published:** 2019-05-28

**Authors:** Ryan S. McClure

**Affiliations:** a Biological Sciences Division, Pacific Northwest National Laboratory, Richland, Washington, USA

**Keywords:** microbiome, multi-omic, network

## Abstract

Within the last decade, there has been an explosion of multi-omics data generated for several microbial systems. At the same time, new methods of analysis have emerged that are based on inferring networks that link features both within and between species based on correlation in abundance. These developments prompt two important questions. What can be done with network approaches to better understand microbial species interactions? What challenges remain in applying network approaches to query the more complex systems of natural settings? Here, I briefly describe what has been done and what questions still need to be answered. Over the next 5 to 10 years, we will be in a strong position to infer networks that contain multiple kinds of omic data and describe systems with multiple species. These applications will open the door for a better understanding and use of microbiomes across a variety of fields.

## Abstract (precis)

Within the last decade, there has been an explosion of multi-omics data generated for several microbial systems. At the same time, new methods of analysis have emerged that are based on inferring networks that link features both within and between species based on correlation in abundance.

## PERSPECTIVE

## GENE COEXPRESSION NETWORKS

Over the last several years, there has been a rapid increase in the depth, cost efficiency, and use of high-throughput omics techniques that can query the abundance levels of a large number of biological molecules simultaneously. These include amplicon analysis, transcriptomics, proteomics, and metabolomics, among others. The collection of these data can answer many questions focused on responses of organisms to specific conditions. However, when experiments collect tens to hundreds of omics data sets, analysis can take the next step and use these data to infer feature coexpression networks. While the specific methods vary, all such networks are essentially based on linking pairs of features (with a feature being either a species, transcript, protein, metabolite, etc.) based on their correlation in abundance (i.e., expression) across a range of variable environmental conditions under which the omics data have been collected. Once inferred, such networks can show which features are coexpressed and can reveal a number of characteristics of the system under question, including how features are related to each other in their expression or abundance, which functional processes are coordinated, which features occupy central positions in the network and are thus important for the growth and bioactivity of the system, which transcripts/proteins act in regulatory pathways, and what the putative function of uncharacterized genes may be. Coexpression networks have been inferred for a number of different systems and have been an especially critical tool in examining human disease (1–3).

## USING GENE COEXPRESSION NETWORKS TO EXAMINE CYANOBACTERIAL SYSTEMS

In our work, I have used network approaches with several different species with the greatest focus on bacterial systems. I have published a number of studies using network analysis to query a model cyanobacterial species, *Synechococcus* 7002. In one set of experiments, I collected transcriptomic data for *Synechococcus* 7002 under 42 separate short-term growth experiments and inferred a network using a mutual information-based tool termed Context Likelihood of Relatedness (CLR). The resulting network of several hundred nodes (transcripts) and edges (instances of high coexpression between transcripts across conditions) was used to identify new targets of protein and RNA regulators in this organism and view how specific processes were coordinated during growth (4). I also expanded this analysis by identifying strong overlap between those genes that have high centrality (i.e., occupy well-connected positions) in the network and those that are critical for growth and metabolism (5). Centrality can be defined by a number of characteristics, with the two most common being degree (the number of edges a node has with other nodes) and betweenness (a measure of how important a node is in connecting two larger clusters of nodes). Nodes of high centrality can be thought of as keystone nodes, as their removal would have deleterious effects on network structure. The linking of network centrality to general importance has also been seen with networks of other systems, including human pathogens, with analysis identifying a strong association between central position in a network and involvement of the gene in infection and virulence, and human cancer data (2, 3, 6). Regarding networks involving more than one species, these have for the most part been limited to species coabundance networks. Such networks have been powerful tools used to identify important keystone species, including in systems related to human health (7, 8). However, taking the next step and linking specific genes across these species, analogous to the way that genes within a species can be linked as described above, is a field that is still relatively unexplored.

## EXPANDING NETWORKS TO MULTIPLE SPECIES

One of the most powerful aspects of gene coexpression networks is the ability to link genes, based on their coexpression, that may be distantly separated on the physical genome. While other approaches to find related genes have focused on physical proximity, coexpression network analysis is location agnostic and can link related genes without this information, indeed in spite of the fact that related genes may be spread across the genome. As network approaches have been used to find gene pairs that are involved in related processes but are physically distant, our group reasoned that this approach could also be applied to systems of multiple species and would be able to link genes that are separated into distinct species but are involved in related processes such as division of labor or metabolic coordination.

## APPLICATION OF GENE COEXPRESSION NETWORKS TO MULTISPECIES SYSTEMS

To apply gene coexpression analysis to multiple species, I collected a large amount of transcriptomic data for two species grown in coculture. These species consisted of an autotropic cyanobacterium, Thermosynechococcus elongatus BP1, and a heterotrophic species, Meiothermus ruber strain A. I cultured these species together under 25 separate short-term perturbations and collected metatranscriptomic data, covering the transcriptome of each species, after each perturbation. For nearly all the perturbations collected, no exogenous sources of organic carbon or nitrogen were added. This means that during growth *M. ruber* was completely dependent for these nutrients on *T. elongatus*. The dependency of *M. ruber* on *T. elongatus* leads to a large amount of interaction and sharing of resources between these organisms during growth. Using the collected transcriptomic data and the CLR program, I inferred a network that linked transcripts between each of these species based on their coexpression. Subsequent analysis of this network revealed that there was a large amount of transcript coordination between the two species that centered on amino acid production, nitrogen metabolism, terpenoid metabolism, and cellular motility (9). By focusing on centrality, I found that these processes occupied much more central (i.e., important) positions in a network comprised of transcripts from both species compared to networks inferred for each species alone. The increased position of these genes indicates greater importance for these processes during coculture and suggests that they are critical for the interactions between these species. This study was one of the first that used network analysis to look at multispecies bacterial systems, and this approach identified several processes, as well as specific genes, that may be crucial for phototroph-heterotroph interactions.

Networks that link genes across species based on coexpression are only beginning to be inferred. Two additional recent studies have used them to examine host-pathogen interactions. One study looked at multispecies networks to link processes in Aspergillus flavus and its host Zea mays and another inferred a network looking at Candida albicans during invasion of immune cells of mice (10, 11). By linking genes across species, these studies inferred where there are pathogenic responses of an infecting organism coordinated with immune responses of the host. Uncharacterized genes that show strong coordination across species may point to new aspects of pathogenicity or immune response, opening the door for the development of novel treatment regimens for several diseases.

## INTERROGATING NETWORKS FOR BIOLOGICAL INSIGHT

One of the major challenges with network analysis, particularly when multiple species are involved, is drawing a distinction between edges in a network that point to actual instances of interaction, cross talk, or metabolite exchange between species and edges that merely reflect a similar response between two genes across conditions. Answering this question will allow for a much greater amount of relevant biological information to be gleaned from a network analysis that examines multiple species. I have applied several different analytical techniques to such networks to try to highlight which edges may point to true interactions. First, as metatranscriptomics usually requires a metagenome (though *de novo* assembly is possible), some information about the genomic potential of each constituent of a multispecies system can be gained. Edges that link processes that are involved in biosynthesis of certain metabolites and vitamins in one species (metabolic pathways) with processes that are involved with obtaining these molecules in another species (transport and uptake systems) likely point to true interactions. In this scenario, one species makes a molecule and the other takes it up. Networks that can identify these instances of correlation are powerful ways to generate strong hypotheses for downstream experiments, possibly using labeled carbon sources or tagged metabolites to track the flow of carbon and nitrogen through a community, ideally from one species to another as the network predicts.

## COMBINING MULTIPLE COEXPRESSION MEASUREMENTS IN A SINGLE NETWORK

I have also looked at the direction of correlation of the gene pair linked by an edge. While mutual information does not provide this information, other analyses such as Pearson or Spearman correlation coefficient can. By looking at similar processes whose genes are negatively correlated between two species, it is possible to identify which processes might be primarily carried out in one organism with the products being shared with other species in the system. The downregulation of certain metabolic processes in one species while another species upregulates these same processes points to this sharing. It is energetically more favorable to obtain nutrients from a partner species than synthesize them yourself. Another approach that other researchers have explored is the collection of time-series data to infer the sequence of events within interactions. The biosynthesis of a needed metabolite by one species preceding the increased expression of the uptake processes for this metabolite by another species is stronger evidence of interaction and sharing than the observation of both of these events lacking a time element (12). Other studies have also looked at metabolic pathways in microbial systems by linking species to metabolites to identify which metabolites may be related to certain microbial community structures (13).

## CHALLENGES WITH INFERRING NETWORKS OF MULTI-OMIC DATA

Delineating true interactions from network noise is one challenge facing multispecies networks. Another is the incorporation of omics data from multiple species into a single network. The response of a community is comprised of contributions from each constituent member of the community with the interactions between them being critical to the community response. Because of this, networks that model community behavior should include as many interactions as possible from different species. However, our group has found that inferring networks that include, for example, transcriptomic data from multiple species often leads to very segregated networks with clusters of transcripts from each species separated from each other and very few edges linking transcripts across species. I have explored a number of alternative network inference tools aside from CLR and Pearson correlation coefficient and have found that a random forest approach, GENIE3, is particularly well suited to inferring networks that link transcripts across species with no loss in overall network robustness or accuracy. Other researchers have also used this approach for interrogating networks, and studies that rank network inference tools have found that GENIE3 is the most accurate choice when linking Escherichia coli target and regulator pairs (14). Whether GENIE3 is the proper tool for multispecies network inference generally remains to be seen, but inferring integrated networks that better link species within a microbial community will be crucial to creating the best models possible of these systems. The observation that GENIE3 is well suited to creating integrated networks suggests that researchers may want to focus on random forest inference methods when building networks of multispecies systems or, at the very least, explore several inference methods before deciding on an approach, as there appears to be great variability in how inference methods fit characteristics of a certain experiment (multispecies, multi-omic, proteomics, transcriptomics, etc.). The strong segregation of edges in multispecies systems also suggests that when looking for correlation between species, these instances of cross-species correlation may be “drowned out” in effect by much higher levels of correlation between genes within a species. Whole-scale ranking of all instances of correlation (both those within and those across species) may miss certain correlations that are high compared to other correlations across species but low compared to correlations within species. Ranking each of these types of correlations separately may be a better strategy for predicting interspecies interactions.

## CONCLUSIONS AND FUTURE DIRECTIONS

Interspecies interactions within microbiomes are extremely complex, and identifying interactions and understanding their contribution to the overall community can be very difficult. However, as community response is a culmination of specific interactions, their delineation is critical. With the large increase in the availability of omics analysis, we now have the tools to use network inference to predict and understand interspecies interactions in natural microbial communities. Some of my own work and other recent studies have shown that such networks are powerful ways to view how processes are related between species. They can predict where metabolites might be shared between species or where there may be instances of division of labor and metabolic coordination by looking for instances of coordinated production and uptake. Through the application of centrality, they can also be used to identify genes, and thus processes, that are particularly important to a system. The success of networks that have been seen with simple systems (phototroph/heterotroph, host/pathogen) shows the utility of this approach, and by understanding the challenges and where the pitfalls are, we are well positioned as a community to apply network techniques to more complex systems comprised of many species. Moving forward, it will be critical to ensure that the right network method is being applied to the right experiments. We can see here that many inference approaches have particular strengths over others, and the correct application of these approaches will be needed to gain the most robust insight into the systems we are interrogating. As we continue to apply network approaches to more complex systems, the hurdles of these more species-rich networks will likely require a greater incorporation of metagenomics and metabolomic data, either knowledge of certain pathways (KEGG databases) or the collection of metabolomics, to query what molecules are present. Over the next decade, I expect to see several new network studies that specifically attempt to link not only species but specific transcripts and proteins across species in complex systems such as soil, marine environments, or human anatomical sites. The increase in metagenomic data as well as critical work being done on how to use network inference tools for these complex multispecies systems will make multispecies networks easier to infer, interrogate, and interpret. The very detailed and specific knowledge that multispecies networks can offer opens up the possibility for increased understanding of a number of microbiomes, allowing us to modify and harness them to improve human health and wellbeing.
